# Temporal transcriptomics identifies early-response and infection-condition-specific modules guiding host-directed anti-EBOV therapeutics

**DOI:** 10.1128/spectrum.03608-25

**Published:** 2026-04-30

**Authors:** Nailou Zhang, Shaolong Zheng, Xiaoxiao Gao, Dan Chen, Wen Xu, Jinbo Wei, Qingwen Ding, Wujian Li, Sandra Chiu, Zhenhua Zheng

**Affiliations:** 1 State Key Laboratory of Virology and Biosafety, Center for Emerging Infectious Diseases, Wuhan Institute of Virology, Center for Biosafety Mega-Science, Chinese Academy of Sciences 74614 , Wuhan, China; 2 National Biosafety Laboratory, National Virus Resource Center, Chinese Academy of Sciences, Wuhan, China; 3 Department of Laboratory Medicine, the First Affiliated Hospital of USTC, Division of Life Sciences and Medicine, University of Science and Technology of China 12652, Hefei, Anhui, China; 4 Division of Life Sciences and Medicine, University of Science and Technology of China 12652, Hefei, Anhui, China; 5 Key Laboratory of Anhui Province for Emerging and Reemerging Infectious Diseases, Hefei, Anhui, China; Karolinska Institutet, Stockholm, Sweden

**Keywords:** Ebola virus, temporal transcriptomics, systems medicine, host-directed antivirals, condition-specific co-expression networks

## Abstract

Ebola virus (EBOV) is among the most lethal human pathogens, yet effective treatment options remain limited. While extensive efforts have elucidated the functions of viral proteins, the temporal orchestration of host transcriptional responses—and their exploitation by EBOV during infection—remains poorly defined. Here, we performed integrated time-series transcriptomic profiling using both RNA-seq and microarray analyses to characterize dynamic host and viral gene expression. Through the combination of co-expression network construction and causal inference modeling, we reconstructed the sequential activation of viral and host genes across distinct phases of infection. Our findings show that EBOV induces only minimal transcriptional perturbations during early infection but extensively reprograms host gene expression at later stages, forming infection-specific co-expression modules enriched for antiviral signaling, immune regulation, and stress responses. To prioritize functional host factors, we integrated transcriptional modules with virus-host protein-protein interaction networks and gene-drug databases. This approach identified early-induced host genes with known or predicted interactions with EBOV proteins and pharmacologically actionable targets. RNA interference-mediated silencing of three key regulatory genes (RELB, LDLR, and MYC) significantly impaired EBOV RNA replication and progeny virus production. Furthermore, pharmacological screening identified sorafenib and thioguanine as effective inhibitors of EBOV replication, with EC₅₀ values of 1.529 μM and 2.469 μM, respectively. Collectively, our study uncovers temporally resolved host regulatory programs hijacked by EBOV and demonstrates the utility of integrating dynamic transcriptomics with systems biology, functional validation, and drug screening to identify host-targeted antivirals. These findings provide a conceptual and methodological framework for developing host-targeted therapies against highly pathogenic viruses.

**IMPORTANCE:**

Ebola virus is a devastating pathogen with limited treatment options. A major challenge in developing therapies is understanding how the virus dynamically hijacks our cells over time. This study provides a time-resolved map of the host transcriptional landscape during Ebola virus infection. We reveal that the virus causes minimal early changes but extensively reprograms human gene expression later, creating specific co-expression networks that are essential for viral replication. By integrating these networks with virus-host interaction data, we identified key human genes and demonstrated that silencing them impairs viral replication. Furthermore, we repurposed existing drugs, identifying sorafenib and thioguanine as effective inhibitors. Our work uncovers the temporal strategy of Ebola virus and establishes a framework for discovering host-directed therapies against this and other highly pathogenic viruses.

## INTRODUCTION

Ebola virus (EBOV), a negative-sense RNA virus of the Filoviridae family, causes severe hemorrhagic fever with high mortality and epidemic potential (1–3). Since its identification in 1976, outbreaks, including the 2013–2016 West African epidemic and the 2025 Sudan virus outbreak in Uganda, highlight its ongoing threat (2, 4). Despite advances in understanding viral entry via the NPC1 receptor and therapeutic development (5–7), supportive care remains central, and universally effective antivirals are lacking (6). A comprehensive understanding of EBOV infection dynamics is currently incomplete, making it critical for developing host-targeted interventions.

EBOV infection involves tightly coordinated virus-host interactions. Viral glycoprotein (GP) mediates host cell attachment and internalization; endosomal proteolytic cleavage of GP then exposes its NPC1-binding domain to facilitate membrane fusion (8–12). Viral proteins VP35 and VP24 antagonize innate immunity by inhibiting RIG-I signaling and STAT1 nuclear translocation, delaying host detection (13). Beyond intrinsic viral mechanisms, successful infection requires remodeling of host metabolism and signaling to create a replication-permissive environment (14–17). EBOV enhances lipid biosynthesis and cholesterol homeostasis to support membrane formation, virion assembly, and budding (18–20). Host factors involved in these pathways, such as endolysosomal cholesterol regulators and NPC1-interacting proteins, are promising therapeutic targets (5, 21, 22).

Time-resolved transcriptomics captures dynamic virus-host interactions, facilitating identification of causal relationships and stage-specific regulatory programs (23–29). Static data sets fail to capture these sequential events, limiting our ability to understand infection progression (30–35).

In this study, we used time-series transcriptomics to characterize dynamic host responses in EBOV-infected Huh7 cells. We introduce a “molecular trigger to system-wide remodeling” framework to capture temporal regulation of host responses. This framework reveals the sequential activation of viral and host genes, identifies infection-specific co-expression modules critical for viral replication, and integrates module-key-node analysis to prioritize essential host factors and candidate antivirals. Our approach combines temporal modeling with functional validation, providing a systems-level understanding of EBOV pathogenesis and uncovering potential host-directed therapeutic strategies.

## RESULTS

### EBOV infection dynamics: Minimal early transcriptional perturbations, expansive late-stage changes

**Fig 1:**
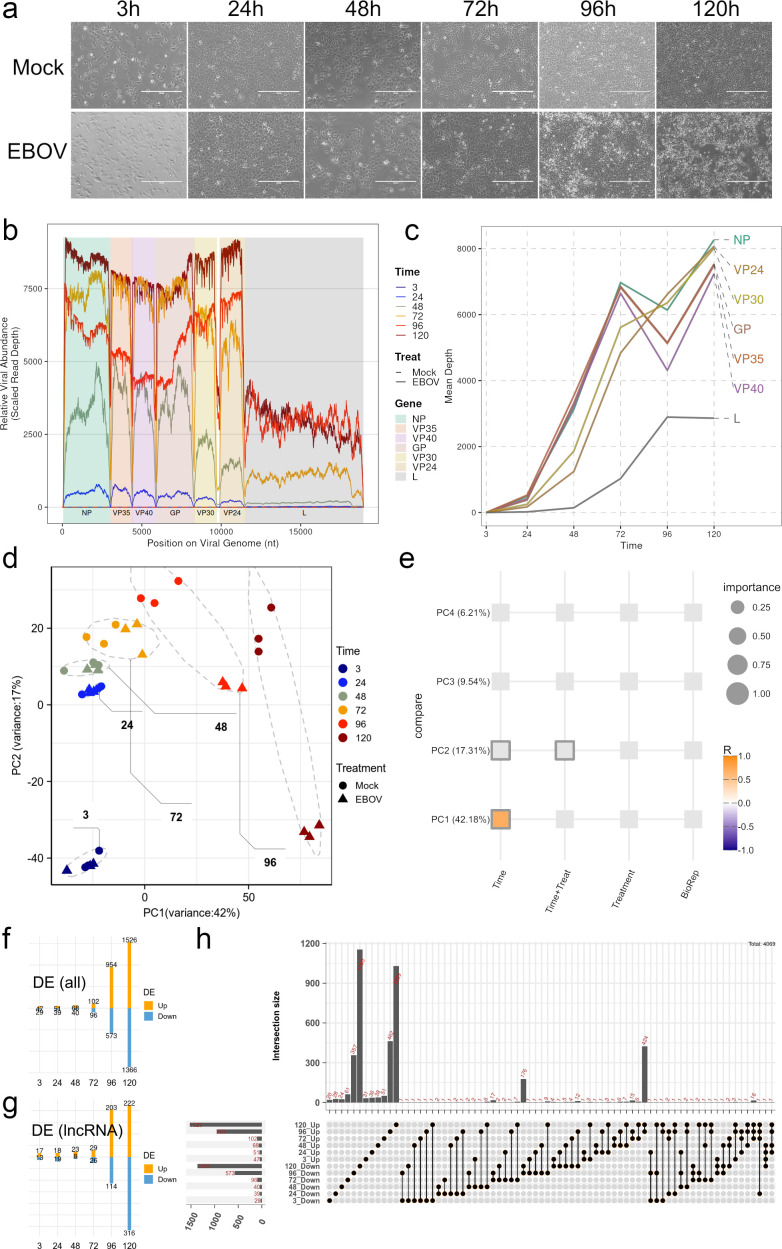
Coordinated expression dynamics of host and viral genes during EBOV infection. (**a**) CPE of EBOV-infected and mock-infected Huh7 cells over time (microscopy images). (**b**) Genome-wide distribution of viral RNA abundance across the EBOV genome at different time points. The x-axis represents the nucleotide position along the viral genome (nt), with gene regions (NP, VP35, VP40, GP, VP30, VP24, and L) indicated by shaded background segments. The y-axis shows relative viral RNA abundance, normalized sequencing read depth by library size. Colored lines correspond to different time points (3, 24, 48, 72, 96, and 120 hpi), as indicated in the legend. Mock samples show negligible viral signal, while EBOV-infected samples display time-dependent accumulation of viral transcripts. (**c**) Temporal changes in mean expression levels of individual EBOV genes. The x-axis indicates time post-infection (hours), and the y-axis represents the mean sequencing depth (average read coverage) for each viral gene. Each colored line corresponds to a specific viral gene (NP, VP24, VP30, GP, VP35, VP40, and L), as labeled on the right. This panel highlights gene-specific differences in transcriptional dynamics over time. (**d**) Principal component analysis (PCA) of host transcriptomes. Each point represents a sample, colored by time point and shaped by treatment (Mock vs EBOV). The axes indicate the first two principal components (PC1 and PC2) with explained variance percentages. (**e**) Variance partitioning analysis showing the contribution of experimental factors (Time, Treatment, Time × Treatment, and Biological replicate) to gene expression variability. Dot size indicates relative importance, and color represents the direction and magnitude of correlation (*R* value). (**f**) Number of differentially expressed genes (DEGs) at each infection time point; orange indicates upregulation, light blue indicates downregulation. (**g**) Distribution of differentially expressed polyadenylated lncRNAs at each infection time point; color scheme as in panel f. (h) UpSet plot showing intersections of DE genes across time points. The upper bar plot shows the number of genes in each intersection, and the matrix below defines the specific combinations of time points. Detailed gene expression values and lists of differentially expressed genes are provided in Table S1.

To investigate host and viral gene expression dynamics during EBOV infection, Huh7 cells were infected at a multiplicity of infection (MOI) of 0.01. Samples were collected at 3, 24, 48, 72, 96, and 120 hours post-infection (hpi) for high-throughput sequencing. Cellular morphology and cytopathic effects (CPE) were documented through microscopy (Fig. 1a). No significant morphological changes were observed in the early phase (3–48 hpi). By 72 hpi, moderate CPE became evident, progressing to severe cellular damage at the late stage (96–120 hpi, Fig. 1a). Viral reads from sequencing data were mapped to the EBOV genome to assess per-base coverage depth. Normalization using DESeq2, based on library size, ensured comparability across samples. The majority of reads were aligned to coding regions, while intergenic regions exhibited relatively lower coverage (Fig. 1b), consistent with previously reported transcriptional patterns of filoviruses (36). EBOV genes exhibited three distinct expression patterns. NP, GP, VP35, and VP40 followed an exponential increase before 72 hpi, reaching peak expression at this time point (Fig. 1b and c). Their expression subsequently declined, reaching a minimum at 96 hpi, followed by a secondary increase (Fig. 1b and c). VP24 and VP30 displayed a continuous upregulation pattern throughout the infection period (Fig. 1b and c). The L gene showed a slow increase until 72 hpi, peaked at 96 hpi, and remained stable thereafter (Fig. 1b and c). By the late stage of infection, NP, GP, VP35, VP40, VP24, and VP30 converged to similar expression levels, whereas L gene expression remained consistently 2–3 times lower than that of other viral genes (Fig. 1c). These findings highlight distinct transcriptional regulation patterns of EBOV genes and provide insights into the temporal dynamics of viral replication.

To investigate host gene expression alterations induced by EBOV, we performed PCA on normalized gene expression profiles (Fig. 1d). Time accounted for the largest proportion of transcriptional variance, explaining 42.18% of the total (Fig. 1d and e). As infection advanced, EBOV-infected samples gradually diverged from mock controls over time (Fig. 1d). Despite this temporal shift, the direct effect of viral infection on global host gene expression remained limited (Fig. 1e). We next conducted differential expression analysis between infected and control samples at each sampling interval, using a fold change threshold >1 and *P*-value <0.05. Prior to 72 hpi, fewer than 100 genes exhibited significant changes, indicating a mild early response. A marked increase in DEGs was observed only during late infection (96–120 hpi), with hundreds to over a thousand genes showing altered expression (Fig. 1f). Approximately 25%–33% of these DEGs were annotated as long non-coding RNAs (polyadenylated lncRNAs) (Fig. 1f and g). Most DEGs were uniquely associated with specific sampling intervals, showing minimal overlap across stages (Fig. 1h). Only a few genes displayed consistent expression patterns throughout the infection course, and concordant regulation was observed predominantly in late-stage infection (96–120 hpi, Fig. 1h). These findings indicate that host transcriptional responses to EBOV are temporally stratified and phase-specific. In contrast to the limited changes observed during early infection, late-stage infection triggered a robust and coordinated host response, underscoring a delayed but pronounced host reaction to viral replication and CPE.

**Fig 2:**
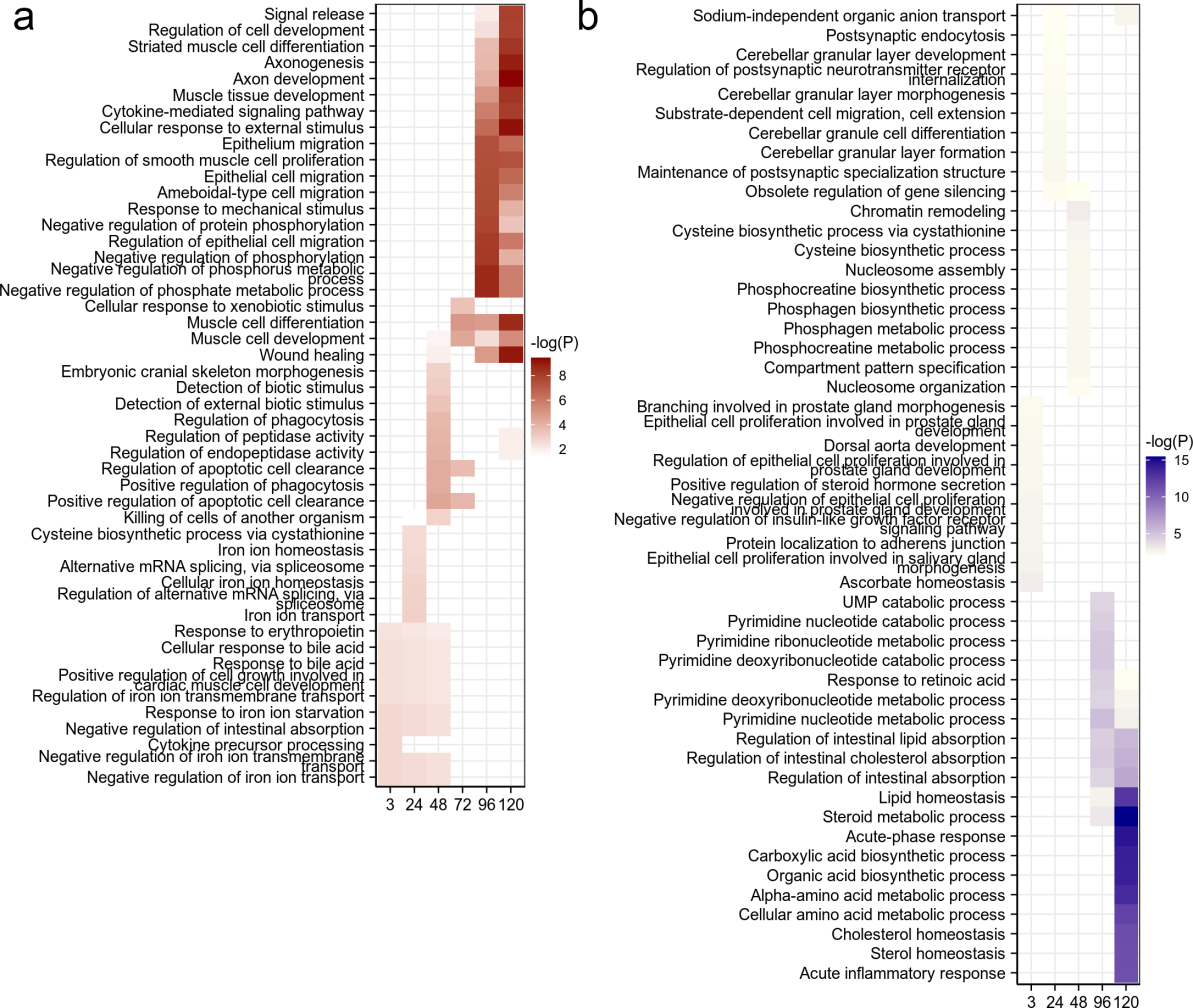
Time-resolved enrichment of GO: Biological process terms in differentially expressed genes during EBOV infection. (**a**) Enriched GO:BP terms among upregulated genes at each infection time point. (**b**) Enriched GO:BP terms among downregulated genes at each infection time point.

To elucidate the biological functions associated with host transcriptional responses, we performed gene ontology (GO; Biological Process, GO:BP) enrichment analysis on DEGs at each sampling interval. The top 10 enriched terms were identified and analyzed for each infection stage (Fig. 2). Clear temporal specificity in functional enrichment patterns was observed. During the early phase of infection (3–48 hpi), upregulated DEGs were primarily enriched in terms such as iron ion homeostasis, cytokine precursor processing, and response to bile acid, suggesting an immediate but limited innate response and metabolic modulation. At the intermediate stage (72 hpi), upregulated DEGs were enriched in detection of biotic stimulus, reflecting the initiation of pathogen recognition processes (Fig. 2a). In the late phase (96–120 hpi), functional terms such as signal release and cytokine-mediated signaling pathway became dominant, indicating a broad activation of immune and intercellular signaling pathways (Fig. 2a). In contrast, downregulated DEGs exhibited stronger temporal segregation in their functional profiles (Fig. 2b). Prior to 48 hpi, no overlapping GO terms were enriched across different sampling points, suggesting minimal functional convergence during early suppression (Fig. 2b). However, in the late stage (96–120 hpi), pronounced repression was observed in pathways related to lipid metabolic homeostasis and cholesterol-associated processes, indicating a host metabolic shutdown likely driven by sustained viral replication and cellular stress (Fig. 2b). Collectively, these results reveal that EBOV infection elicits stage-dependent functional remodeling of the host transcriptome. While early responses are limited and diverse, late infection induces a coordinated activation of immune pathways and repression of metabolic functions.

### Causal inference reveals early-activated gene module as a target for anti-EBOV drugs

**Fig 3:**
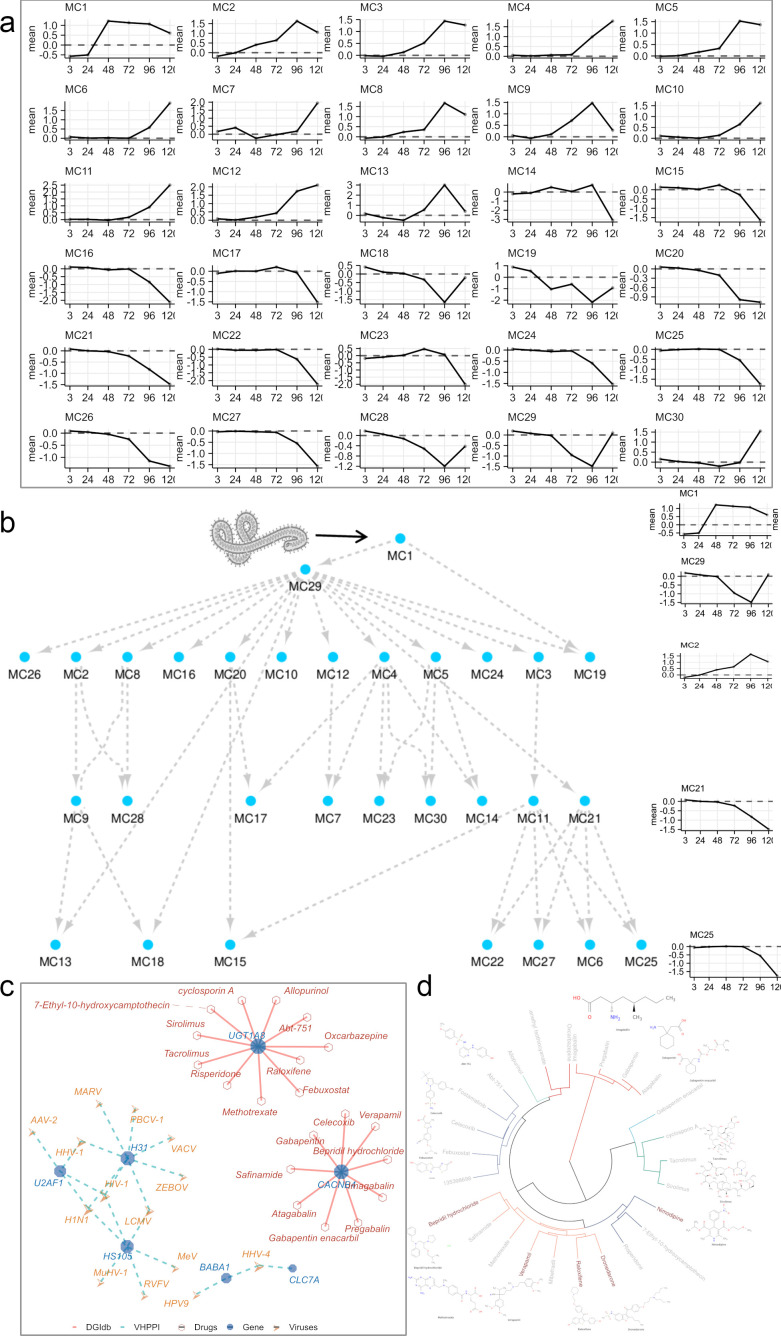
Regulatory hierarchy of EBOV-induced host gene modules. (**a**) Line plots showing the average log₂ fold change of genes within each co-expression module across infection time points. (**b**) Regulatory relationships among host gene modules inferred using causal structure inference, revealing sequential activation patterns. (**c**) Tripartite network connecting virus, host genes, and known drug molecules based on HVIDB and DGIdb. (**d**) Clustering of candidate compounds based on molecular fingerprint similarity. Compounds labeled in red represent known EBOV inhibitors. The complete list of genes included in each co-expression module is provided in Table S2.

To capture the dynamic transcriptional responses to EBOV infection, we employed splineTimeR, a natural cubic spline-based regression framework. Unlike threshold-based differential expression (DE) analyses that detect changes at discrete time points, splineTimeR models gene expression trajectories across the entire infection timeline. Genes with distinct temporal expression patterns between EBOV- and mock-infected samples were clustered into 30 co-expression modules using Mfuzz, a fuzzy c-means algorithm based on normalized expression profiles. Average differential expression between EBOV-infected and mock-infected groups was computed and visualized for each module across the infection timeline. Three distinct temporal expression patterns emerged (Fig. 3a): Pattern 1. Monotonic late-phase regulation—Genes showed minimal changes during early infection, followed by sustained up- or downregulation from mid to late phases (e.g., MC4, MC6, MC16, MC20–MC22, MC24–MC27).; Pattern 2. Transient activation or suppression—A sharp peak or trough around 96 hpi, followed by gradual increase or decrease (e.g., MC2, MC3, MC5, MC8, MC9, MC28, MC29); Pattern 3. Phase-reversal dynamics—Early downregulation followed by late upregulation, or vice versa (e.g., MC1, MC2, MC18, MC19, MC28). Gene ontology enrichment analysis revealed that immune-related biological processes were predominantly enriched in modules MC2, MC4, MC8, MC15, MC17, MC22, and MC27 (Fig. S1). These immune modules followed expression patterns 1 and 2, indicating that host immune responses were predominantly regulated during the mid-to-late stages of EBOV infection.

Temporal differences in module activation were observed. For instance, MC1 genes diverged earlier and more sharply from baseline than MC2, suggesting a leading role in the early host response. To further explore these regulatory delays and infer causality between modules, we applied Causal Structure Inference (CSI) analysis (https://github.com/cyversewarwick/csi), a time-lagged network inference method. CSI predicted that MC1 functions as an early-response hub that drives downstream repression of MC29 and sequential activation or suppression of additional modules (Fig. 3b). This cascade aligns with the observed expression slopes and timing, supporting the utility of CSI in reconstructing transcriptional hierarchies post-infection.

Given its role as the earliest responsive module, MC1 was examined in detail. MC1 comprised 79 genes, including 39 protein-coding genes, 25 lncRNAs, and 15 pseudogenes. Integration with the Human Viral Interaction Database (HVIDB) database identified multiple MC1 proteins that interact with viral components (Fig. 3c). U2AF1, H3C8/H31, HS105, BABA1, and CLC7A engage in direct interactions with multiple viral proteins (Fig. 3c). Among these, H3C8 (UniProt: P68431) is shown to bind Zaire ebolavirus VP24 (UniProt: Q05322) (37), a key immune evasion protein. H3C8 is involved in RNA polymerase I promoter opening and pre-replication complex assembly.

Drug-gene interaction data from DGIdb highlighted CACNB4 and UGT1A8 as potentially druggable MC1 targets (Fig. 3c). CACNB4, a subunit of voltage-gated calcium channels, interacts with several approved drugs, including verapamil, nimodipine, dronedarone, and bepridil hydrochloride—all previously reported to exhibit anti-EBOV activity (38–40). CACNB4 regulates calcium ion influx, a critical step for EBOV entry via two-pore channels (TPCs) in the endosome. Verapamil inhibits TPC-mediated calcium release, thereby impairing viral trafficking and infection (38). UGT1A8 encodes a glucuronosyltransferase involved in steroid metabolism and interacts with raloxifene, a selective estrogen receptor modulator with reported anti-EBOV activity (41). Although direct mechanistic links between these drugs and gene-specific inhibition of EBOV remain to be validated, early response and druggability suggest MC1 as a promising source of host-directed therapeutic targets.

To explore shared chemical features among active compounds, we performed chemical fingerprint clustering of 20 small molecules targeting MC1 genes (Fig. 3d). Verapamil, raloxifene, dronedarone, and bepridil formed a structural cluster characterized by a phenyl ring coupled to a long aliphatic chain (Fig. 3d). This common scaffold may inform rational design of future anti-EBOV agents.

In summary, dynamic modeling uncovered distinct temporal expression programs in response to EBOV infection. MC1 emerged as an early-response module potentially orchestrating downstream transcriptional changes. Combined analysis of transcriptomics, virus-host interactions, and drug-target associations highlights MC1 as a key regulatory node and a reservoir of potential therapeutic targets for host-based antiviral strategies.

### EBOV infection remodels the host transcriptomic landscape

Although temporal activation patterns of host genes shed light on virus-induced transcriptional reprogramming, successful EBOV infection requires extensive remodeling of host transcriptional, metabolic, and proteostatic networks. Gene modules that are uniquely activated under infection conditions—yet remain uncoordinated or at baseline in controls—may play pivotal roles in virus-host interactions and support viral replication. To identify such virus-dependent co-expression modules, we analyzed publicly available EBOV time-series microarray data sets (GSE65573, GSE69942, GSE80058, GSE80832, GSE86539, and GSE210189) (42). Data sets were selected based on predefined criteria, including the use of human cell model, multiple distinct time points for temporal resolution, a minimum of three biological replicates per condition, and high-quality data (minimal batch effects, robust biological replicate clustering, and time as the predominant driver of transcriptional variation) as assessed by PCA (see Materials and Methods and Fig. S2). Our RNA-seq data were not used for this analysis due to limited early-stage transcriptional changes (fewer than 100 differentially expressed genes). Among the public data sets, only GSE86539 fulfilled our criteria for sampling intervals, replication consistency, and minimal batch effects. This data set profiled Huh7-VP30 cells infected with Ebola-ΔVP30 at 0, 8, 24, 48, and 72 hpi, with four biological replicates per condition.

**Fig 4:**
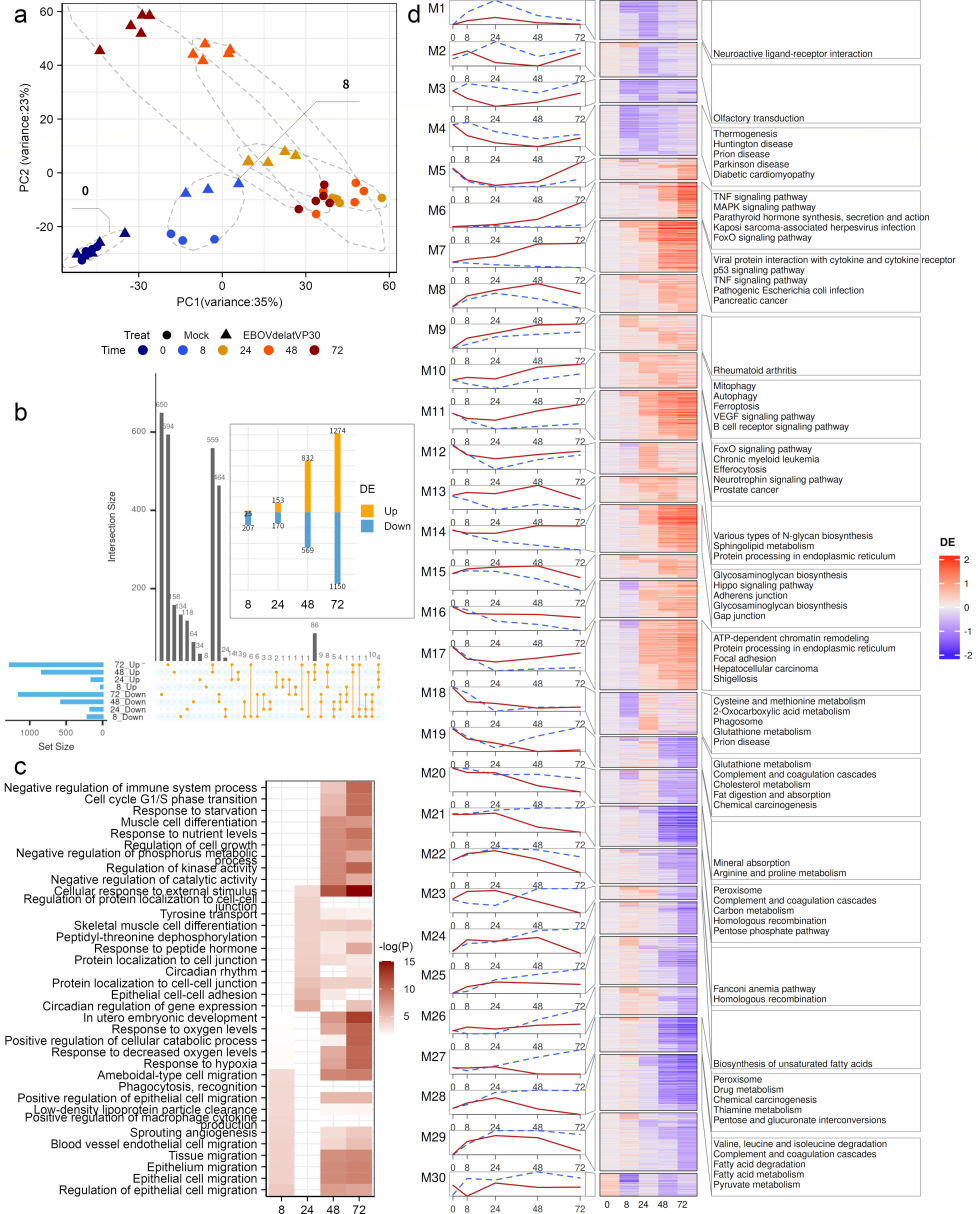
EBOV-ΔVP30-mediated transcriptional reprogramming of Huh7 cells. (**a**) PCA of gene expression profiles in Huh7 cells infected with EBOV-ΔVP30. (**b**) Bar plot and UpSet diagram show the number and overlap of DEGs across time points. Orange indicates upregulation; light blue indicates downregulation. (**c**) GO: Biological process enrichment analysis of upregulated genes at each time point post-infection. (**d**) Co-expression clustering of differentially expressed genes. From left to right: line plots showing average expression of genes in each module under infection (red) and control (blue); heatmaps of log₂ fold change of genes in each module; KEGG pathway enrichment analysis of genes in each module. Gene expression profiles, differentially expressed genes, and co-expression modules derived from the EBOV-ΔVP30 data set are available in Tables S3 and S4.

PCA identified time as the dominant factor driving transcriptional variation (Fig. 4a). Notably, starting at 8 hpi, infected samples gradually diverged from controls along the second principal component (Fig. 4a), reflecting a progressive virus-induced transcriptional remodeling. Differential expression analysis revealed a steady increase in the number of DEGs—from several hundred at 8 hpi to several thousand by 72 hpi (Fig. 4b). Nearly half of these DEGs were specific to individual infection stages (Fig. 4b), supporting our RNA-seq observation of phase-specific gene activation. Conversely, over 100 genes exhibited consistent differential regulation across multiple stages—a pattern that became particularly evident in the late phase, where over 1,000 genes were uniformly regulated (Fig. 4b). Gene ontology enrichment of these DEGs showed significant upregulation of genes involved in the negative regulation of immune system processes, along with sustained activation of pathways related to cellular responses to external stimuli and protein localization to cell-cell junctions (Fig. 4c).

To further resolve these temporal patterns, we applied Mfuzz clustering to the normalized expression profiles, resulting in 30 co-expression modules. Based on their average expression trajectories, the modules could be categorized into four distinct patterns (Fig. 4d): (i) Infection-specific sustained regulation: Modules such as M6 and M7, which show persistent upregulation or downregulation exclusively in infected samples, while controls remain at baseline. (ii) Amplified trends with similar timing: Modules (e.g., M1, M5, M8–M10, M11–M13, M15, M22) displaying similar temporal trends in both infected and control conditions but with increased amplitude under infection. (iii) Temporal shifts in shared expression: Modules like M2 and M16–M21 that exhibit phase shifts or altered oscillation magnitudes between conditions. (iv) Divergent trajectories: Modules (e.g., M3, M4, M16, M23–M30) showing completely distinct expression profiles in infected versus control samples. Functional enrichment analysis indicated that virus-host interaction and immune-related pathways were predominantly enriched in modules M6 and M7, suggesting these gene sets are critical to the viral replication process. In summary, our results reveal that EBOV infection triggers a complex, time-dependent reprogramming of host gene expression. The identification of distinct, infection-specific co-expression modules provides critical insights into the molecular mechanisms underlying the virus.

### Silencing co-expressed genes baselined in mock but activated by infection suppresses viral replication

**Fig 5:**
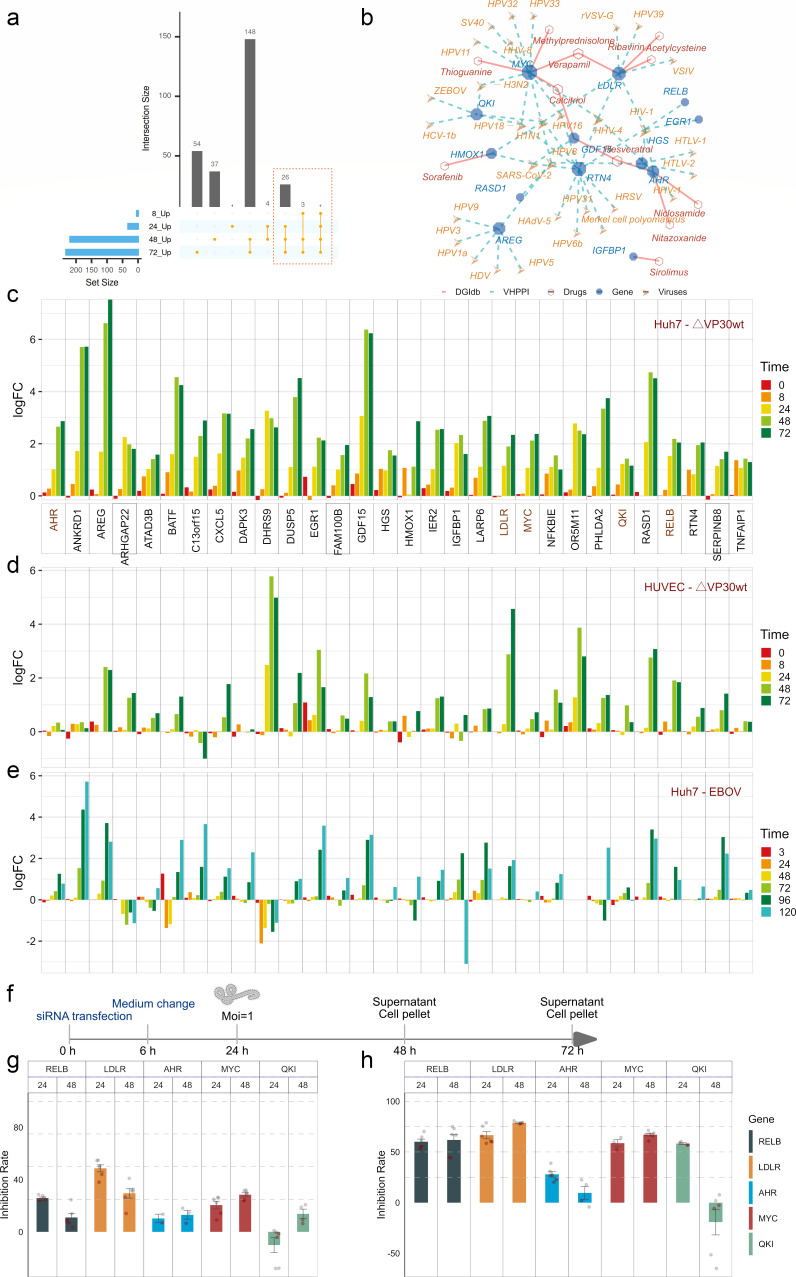
Conserved activation of M7 genes across cell types. (**a**) UpSet plot showing overlap of M7 genes among differentially expressed genes at various time points. (**b**) Tripartite network connecting virus, host genes, and known drug molecules based on HVIDB and DGIdb. (**c–e**) Consistent upregulation of 30 M7 genes across multiple data sets: (**c**) Huh7 cells infected with EBOV-ΔVP30 (microarray); (**d**) HUVEC cells infected with EBOV-ΔVP30 (microarray); (**e**) Huh7 cells infected with wild-type EBOV (RNA-seq). (**f**) Schematic of the experimental workflow: siRNA-mediated knockdown of selected M7 genes followed by assessment of viral replication and progeny production. (**g and h**) Relative inhibition of viral replication in cell lysates (**g**) and supernatants (**h**) following siRNA knockdown, compared to negative controls.

We conducted a detailed analysis of genes within module M7. Differential expression analysis (*P* < 0.05) identified that over half of the 274 genes were significantly upregulated at 48 and 72 hpi (Fig. 5a). Notably, 30 genes showed sustained induction as early as 24 hpi (Fig. 5a), suggesting early and persistent involvement in the host response to EBOV. Virus-host protein-protein interaction mapping using the HVIDB database revealed that nearly half of these 30 genes—including MYC, LDLR, RELB, QKI, HMOX1, GDF15, EGR1, HGS, RASD1, AREG, RTN4, AHR, and IGFBP1—interact with multiple viral proteins (Fig. 5b). In particular, QKI was found to interact with VP30 of Zaire ebolavirus (43), implicating it in a potential virus-specific regulatory role. Drug-target interaction data from DGIdb showed that several of these genes—MYC, LDLR, GDF15, AHR, HMOX1, and IGFBP1—are modulated by existing pharmacological compounds. Among them, LDLR and MYC interact with verapamil (Fig. 5b), an agent with reported anti-EBOV efficacy (38). AHR is linked to niclosamide and nitazoxanide, both with known antiviral activity (44, 45). These observations underscore the therapeutic relevance of this gene subset in the context of EBOV infection.

To determine whether these transcriptional patterns are consistent across different cellular contexts, we compared gene expression in two infection models: EBOV-ΔVP30-infected HUVEC cells (GSE210189) and wild-type EBOV-infected Huh7 cells. Except for ARHGAP22, ATAD3B, DHRS9, and PHLDA2, the majority of genes displayed time-dependent upregulation in both systems, indicating a conserved host response signature (Fig. 5c through e). To examine the functional contribution of RELB, LDLR, AHR, MYC, and QKI, we carried out loss-of-function assays using specific siRNAs in Huh7 cells infected with EBOV at MOI 1 (Fig. 5f). Knockdown efficiency was validated at both transcript and protein levels. Western blotting at 72 hours post-transfection confirmed robust depletion of RELB and QKI proteins and partial but clear reduction of LDLR, AHR, and c-MYC relative to non-targeting control (Fig. S3l). Silencing of LDLR resulted in the most pronounced reduction in viral replication at 24 hpi (inhibition rate 48.67%; Fig. 5g). QKI knockdown showed only marginal effects. In contrast, depletion of RELB, LDLR, and MYC significantly suppressed progeny virus production, achieving inhibition rates of 58%–79% at 48–72 hpi (Fig. 5h). These protein-level reductions directly correlate with the observed antiviral effects, demonstrating that even partial knockdown of key M7 hub genes can substantially impair EBOV replication. This highlights RELB, LDLR, and MYC as critical host factors and promising targets for host-directed anti-EBOV therapeutics.

### Antiviral effects of repurposed compounds identified from infection-specific module M7

Based on the virus-host-drug interaction network within the infection-specific co-expression module M7 (Fig. 5b), we identified calcitriol, sorafenib, resveratrol, methylprednisolone, and thioguanine as candidate compounds. Candidate prioritization was based on high connectivity to key M7 hub genes (MYC, LDLR, AHR, and HMOX1), network centrality, and pharmacological association with EBOV-exploited host pathways for replication and immune evasion. Sorafenib, a multi-kinase inhibitor targeting the RAF/MEK/ERK pathway linked to MYC signaling in M7, inhibits replication of alphaviruses and several other RNA viruses (46, 47). Thioguanine, a purine analog, disrupts nucleotide metabolism essential for viral RNA synthesis and exhibits activity against multiple RNA viruses, including coronaviruses and enteroviruses (48–50). Resveratrol, a polyphenolic compound, exerts broad-spectrum antiviral activity by interfering with viral replication cycles and modulating host inflammatory responses (51). Methylprednisolone and calcitriol were selected due to their network interactions with the M7 hub gene MYC, as annotated in DGIdb. Methylprednisolone, a glucocorticoid, is used in the supportive care of Ebola virus disease to control excessive cytokine responses and inflammation (52), but its direct antiviral activity at the molecular level remains uncharacterized. Calcitriol, the active metabolite of vitamin D, modulates immune responses via vitamin D receptor (VDR) signaling, including the regulation of cytokine production and innate immunity (53). Direct anti-EBOV activity of these five compounds has not been extensively reported in the literature.

**Fig 6:**
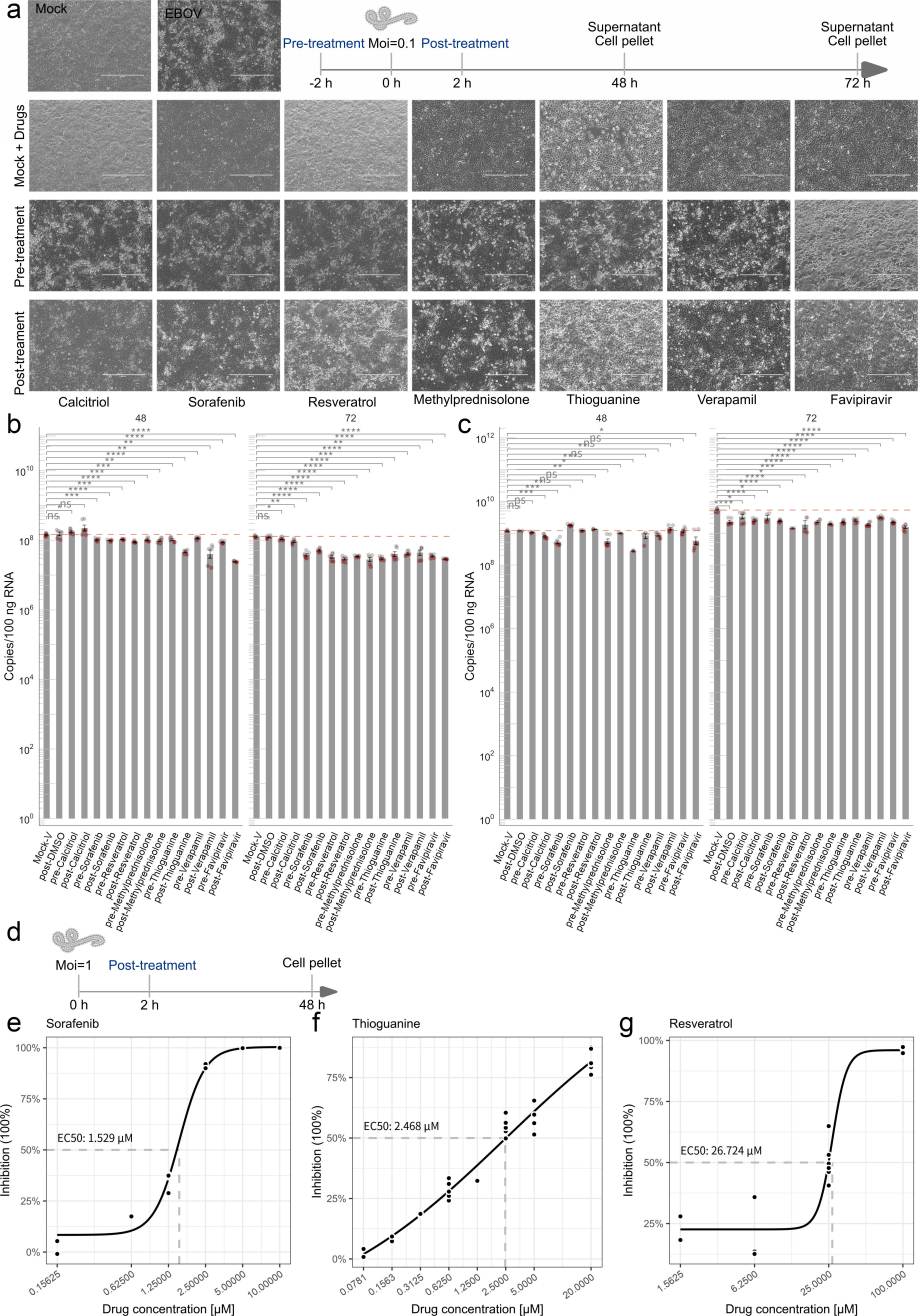
Sorafenib, thioguanine, and resveratrol inhibit EBOV replication in a dose-dependent manner. (**a**) Schematic of experimental design. Representative microscopic images showing CPE at 48 hpi under five conditions: drug-only, virus-only, pre-treatment (drug before virus), post-treatment (drug after virus), and mock-infected control. (**b and c**) Quantification of EBOV RNA copies in cell lysates (**b**) and supernatants (**c**) after drug treatment. Data are presented as mean values, and error bars indicate ±1 standard error of the mean (SEM) (approximately 68% confidence interval). Error bars were calculated in linear space and visualized on a log10-transformed axis. (**d**) Workflow of dose-response experiment to evaluate drug concentration-dependent inhibition of viral replication. (**e, f and g**) Dose-response curves of sorafenib, thioguanine, and resveratrol show inhibition of EBOV replication. Corresponding EC₅₀ values are indicated. Information regarding initial drug screening concentrations and minimum cytotoxic concentrations is provided in Table S5.

To evaluate their antiviral effects, verapamil (host-targeting) and favipiravir (viral-targeting) were included as reference controls for validation. Cells were pre-treated or post-treated with each compound at their maximum non-cytotoxic concentration, followed by infection with EBOV at an MOI of 0.1. Viral RNA levels in both cell lysates and supernatants were quantified by qPCR at 48 and 72 hpi to assess intracellular replication and progeny virus production (Fig. 6a). At 48 hpi, sorafenib and resveratrol most effectively mitigated virus-induced CPE during pre-treatment, indicating a possible protective role on host cells (Fig. 6a). In post-treatment assays, calcitriol, sorafenib, methylprednisolone, and verapamil also reduced CPE to varying extents, whereas favipiravir showed limited protective effects on cell morphology (Fig. 6a). These results imply that some host-targeting compounds may offer better cytoprotection than direct antivirals. Intracellular viral RNA quantification revealed that sorafenib, resveratrol, methylprednisolone, thioguanine, verapamil, and favipiravir all significantly inhibited EBOV replication (Fig. 6b). Calcitriol, however, showed no appreciable effect. By 72 hpi, the inhibitory efficacy of calcitriol, sorafenib, resveratrol, methylprednisolone, and thioguanine was comparable to that of favipiravir, with suppression rates between 59% and 76% (Fig. 6b), indicating sustained antiviral activity. Analysis of progeny virus production further demonstrated that post-treatment with sorafenib, pre-treatment with methylprednisolone and thioguanine, as well as post-treatment with favipiravir, significantly reduced extracellular viral RNA at 48 hpi (Fig. 6c). These results highlight the importance of treatment timing and suggest that both viral and host-directed strategies contribute to antiviral efficacy.

To further validate antiviral potential, we examined the dose-dependent effects of sorafenib, resveratrol, methylprednisolone, and thioguanine in post-infection treatment models (Fig. 6d). A clear dose-response inhibition of viral replication was observed for sorafenib, thioguanine, and resveratrol, with EC₅₀ values of 1.529 μM, 2.469 μM, and 26.726 μM, respectively (Fig. 6e through g). In contrast, methylprednisolone did not exhibit concentration-dependent inhibition, suggesting a non-linear or threshold-limited mechanism. Collectively, these findings validate several repurposed drugs, particularly those with host-modulating properties, as effective inhibitors of EBOV replication. The identification of low micromolar EC₅₀ values for sorafenib and thioguanine underscores their promise for further development as anti-EBOV therapeutics.

## DISCUSSION

Based on temporal transcriptomic and functional analyses, we characterized the host response to EBOV infection as a temporally structured process, which transitions from early transcriptional quiescence to extensive late-stage cellular reprogramming. These findings link dynamic changes in gene expression to regulatory modules and to potential host-directed antiviral strategies.

We characterized EBOV infection dynamics in Huh7 cells using RNA-seq at a low MOI of 0.01. This MOI was specifically chosen to enable multi-cycle viral replication while minimizing rapid CPE in early infection (Fig. 1a). This extended observation period allowed us to capture subtle early transcriptional responses before extensive cell death interfered with transcriptomic profiles. Under these conditions, EBOV genes exhibited distinct expression patterns (Fig. 1b and c). Notably, NP, GP, VP35, and VP40 displayed a biphasic pattern, peaking at 72 hpi, declining, then showing a secondary increase by 120 hpi. This late-stage biphasic expression (Fig. 1b and c), uncommon in single synchronous infections, likely reflects multiple rounds of infection, where progeny virions from initially infected cells re-infect remaining susceptible cells. Dynamic changes in the cellular microenvironment, such as media exhaustion during prolonged culture, may also contribute to these distinct viral gene expression kinetics.

The host transcriptional response at an MOI of 0.01 showed distinct temporal stratification. Early infection (prior to 72 hpi) showed minimal changes, with fewer than 100 DEGs (Fig. 1f). These results are consistent with an early phase of immune evasion by EBOV, during which viral replication proceeds with minimal host perturbation. Early quiescence aligns with EBOV’s immune evasion mechanisms, including VP35-mediated suppression of RIG-I signaling and IRF-3 activation, and VP24 inhibition of STAT1 nuclear translocation (54, 55). As viral load increased, the number of DEGs increased dramatically to over 1,000, which only occurred during late infection (96–120 hpi, Fig. 1f). This surge coincided with the onset and progression of severe CPE (Fig. 1a).

A critical question is whether these significant late-stage transcriptional changes result from specific host-pathogen interactions or are mainly secondary effects of cellular stress and impending apoptosis. We did not include heat-inactivated virus or non-lethal stressor controls; however, several observations argue against these changes being solely due to non-specific cell death. The complex and active viral gene kinetics, including the secondary rise in several viral transcripts (Fig. 1b and c), underscore ongoing viral manipulation, not passive cellular breakdown. Our GO analysis revealed highly time-dependent and functionally specific host transcriptome remodeling (Fig. 2). Late-stage infection (96–120 hpi) showed broad activation of “signal release” and “cytokine-mediated signaling pathways” (Fig. 2a), coupled with pronounced repression of specific metabolic functions like “lipid metabolic homeostasis” (Fig. 2b). These coordinated, distinct functional shifts are not characteristic of non-specific cell death, but rather indicate specific host responses. These responses may be driven by viral strategies to modulate cellular metabolism for replication or host efforts to defend against infection. EBOV is known to extensively manipulate host processes, and even cell death can be strategically exploited for egress and dissemination (13). Thus, while severe CPE undoubtedly affects the late-stage transcriptome, our findings indicate that the observed changes reflect specific host responses, either shaped by EBOV or arising from the host’s dynamic, albeit ultimately insufficient, defense mechanisms. Future studies with heat-inactivated virus or non-lethal stress controls will help distinguish these specific virus-host interactions from general cellular stress.

Network integration analysis identified candidate antiviral compounds, among which sorafenib and thioguanine showed strong dose-dependent inhibition of EBOV replication. Sorafenib inhibits the RAF/MEK/ERK signaling pathway, a known mechanism for its anticancer activity (46). Activation of the ERK pathway contributes to c-MYC protein stability and transcriptional activity (46). Therefore, sorafenib’s inhibition of this pathway is expected to reduce c-MYC protein levels and suppress its transcriptional activity. c-MYC is a major transcription factor that regulates diverse cellular processes, including nucleotide biosynthesis, lipid metabolism, and pathways governing cell growth, survival, and apoptosis (56). Given the significant upregulation of genes within Module M7, which includes MYC and LDLR (Fig. 5b), and the observed repression of lipid metabolic homeostasis in EBOV-infected cells (Fig. 2b), it is plausible that EBOV co-opts these MYC-driven metabolic and survival programs. Thus, sorafenib likely suppresses sustained M7 activation by targeting the RAF/MEK/ERK/MYC axis, thereby disrupting viral protein synthesis and metabolic reprogramming exploited by the virus. Thioguanine, a purine nucleobase analog, acts as an antimetabolite. After intracellular conversion to its nucleotide form, it depletes the cellular purine nucleotide pool and disrupts *de novo* purine biosynthesis (48–50). EBOV replication relies on host nucleotide pools for RNA synthesis and genome replication. Thioguanine constrains the nucleotide supply, directly impeding EBOV RNA-dependent RNA polymerase activity and genome replication, thereby suppressing viral proliferation. Both compounds also mitigated CPE, suggesting potential cytoprotective effects of host-targeting strategies in our model. Together, these two compounds demonstrate the translational potential of M7-derived targets, as sorafenib targets proliferative signaling upstream of MYC, while thioguanine depletes the nucleotide resources that EBOV hijacks for genome amplification.

This study has several limitations that should be considered. Although Huh7 cells provide a reproducible model, they do not fully recapitulate primary target cells (hepatocytes and macrophages), as cancer-associated alterations in Huh7 cells may affect interferon signaling and metabolic processes. In iPSC-derived hepatocytes, EBOV induces delayed interferon responses in bystander cells and suppresses liver-specific gene expression, features that are not fully captured in Huh7 cells (30). In primary macrophages, EBOV infection triggers rapid NF-κB activation and robust cytokine responses, whereas these innate immune dynamics are attenuated or altered in Huh7 (57). Cross-data set consistency supports our core findings, but validation in primary cells, organoids, or *in vivo* models is essential. Additionally, while gene silencing and drug assays confirm the functional relevance of the identified targets, the precise effects of these targets on specific stages of the viral lifecycle remain unclear.

In summary, EBOV infection regulates temporally coordinated transcriptional programs that link early regulatory events to late-stage host cellular remodeling and support viral replication. By integrating dynamic modeling and functional validation, our study provides a framework for understanding EBOV-host interaction dynamics and advancing the development of host-directed antiviral strategies.

## MATERIALS AND METHODS

### Virus and cells

The EBOV strain (Makona-C07, GenBank accession no. KJ660347.2) was stored at the Wuhan Institute of Virology, Chinese Academy of Sciences. The viruses were propagated in Vero-E6 cells, and all the experiments were conducted within a biosafety level 4 (BSL-4) facility. Human hepatoma Huh7 cells were kindly provided by Prof. Xinwen Chen (Wuhan Institute of Virology, China). These cells were cultured in DMEM (Gibco, cat. no. C11995500BT) supplemented with 10% fetal bovine serum (Vazyme, cat. no. F103-01) and penicillin/streptomycin (Biosharp, cat. no. BL505A). They were maintained at 37°C in a humidified incubator with 5% CO₂ and passaged every 2–3 days. The Vero E6 cell line, derived from African green monkey kidney cells, was obtained from the Preservation Center at the Wuhan Institute of Virology. Virus stocks were propagated in Vero E6 cells, and infectious titers were determined by a TCID₅₀ assay. All experiments using live EBOV were conducted in the BSL-4 facility at the National Biosafety Laboratory in Wuhan, China, which is accredited by the China National Accreditation Service for Conformity Assessment and approved by the National Health and Family Planning Commission of China (58).

### Virus titration

Virus titers were determined using the TCID₅₀ assay on Vero E6 cells. Cells were seeded in 96-well plates. Serial 10-fold dilutions (0.1 mL per well) of virus stocks were applied. Plates were incubated at 37°C with 5% CO₂ for 1 hour. Next, 100 μL of medium containing 2% FBS was added to each well. After 5–7 days of incubation at 37°C in 5% CO₂, CPE were evaluated. Finally, the TCID₅₀ per mL was calculated.

### RNA extraction, cDNA synthesis, and qRT-PCR

Total RNA was extracted from cell pellets using TRIzol Reagent (Invitrogen) according to the manufacturer’s protocol. Cells were lysed in TRIzol and then mixed with chloroform. After a brief incubation, samples were centrifuged at 12,000 × *g* for 15 minutes at 4°C. The aqueous phase was collected, and RNA was precipitated with isopropanol. The RNA pellet was washed with 75% ethanol, air-dried, and dissolved in RNase-free water. RNA quality and concentration were verified spectrophotometrically before further processing. In parallel, viral nucleic acids from supernatants were isolated using the FastPure Viral DNA/RNA Mini Kit (Vazyme, cat. no. RC311-01) following the kit manual. For both sample types, cDNA was synthesized using the FastKing RT Kit (TIANGEN, cat. no. KR118-02) per the provided instructions. For viral quantification, 50 ng of RNA-derived cDNA was amplified in a qRT-PCR containing iTaq Universal SYBR Green Supermix (Bio-Rad) and EBOV-specific primers (GP gene, forward: 5′-GGGAATGGAGTGGCAACTGA-3′; reverse: 5′-GCTGCTGGTAGACACTCACT-3′). The qRT-PCR program on the Bio-Rad CFX system consisted of an initial activation at 95°C for 3 minutes, followed by 39 cycles at 95°C for 10 seconds and 55°C for 45 seconds. Viral copy numbers were determined by comparing threshold cycle (Ct) values to a standard curve derived from serial dilutions (1 × 10³−1 × 10⁸ copies/mL) of plasmids containing EBOV cDNA. For host gene expression analysis, cDNA synthesis for host transcripts was performed as described above. Quantitative PCR was conducted using GAPDH as an internal control under the conditions of 95°C for 3 minutes, followed by 38 cycles at 95°C for 10 seconds and 58°C for 30 seconds. Transcript levels were quantified using the 2*^−^*^ΔΔCt^ method and relative standard curves. Primer sequences for target genes included: MYC (forward: 5′-CGTCCTCGGATTCTCTGCTCTC-3′, reverse: 5′-TCCTCATCTTCTTGTTCCTCCTCAG-3′), QKI (forward: 5′-GGTGTATTAGGTGCGGTGGCTAC-3′, reverse: 5′-GCTCGGTCTGCGGTCACAATC-3′), AHR (forward: 5′-GCCAACATCACCTACGCCAGTC-3′, reverse: 5′-TGCCGCTTGGAAGGATTTGACTTG-3′), LDLR (forward: 5′-GCCTCACAGGTTCCGATGTCAAC-3′, reverse: 5′-TCCTCTCACACCAGTTCACTCCTC-3′), and RELB (forward: 5′-CAACGCTGGGTCCCTGAAGAAC-3′, reverse: 5′-TGTCCCTGCTGGTCCCGATATG-3′).

### Experimental design and MOI selection

For our primary RNA-seq time-course experiments, a low MOI of 0.01 was employed. This MOI enables multi-cycle viral replication while minimizing rapid CPEs during early infection, thereby extending the infection window to capture comprehensive temporal virus-host interaction dynamics and early transcriptional responses. For functional validation (e.g., siRNA knockdown) and drug screening assays, an MOI of 0.1 was used. This slightly higher dose ensures a more robust and synchronized infection, facilitating clearer and more sensitive evaluation of antiviral effects under stronger viral-challenge conditions.

### RNA-seq data set and analysis

Huh7 cells were infected with EBOV at an MOI of 0.01. Infected cells were collected at 3, 24, 48, 72, 96, and 120 hpi for transcriptomic analysis. Poly(A)-enriched RNA libraries were constructed using a paired-end 150 bp strategy and sequenced on the Illumina NovaSeq 6000 platform. The poly(A) enrichment protocol specifically selected for polyadenylated transcripts, including messenger RNAs (mRNAs) and polyadenylated long non-coding RNAs (lncRNAs). Raw RNA-seq reads were processed using the *nf-core/rnaseq* pipeline (59). Reads were mapped to the human genome (GRCh38) with the following parameters: *nextflow run nf-core/rnaseq -input samplesheet.csv -fasta Genome.fa -gtf GtfFile.gtf -star_index STAT_directory*. Gene expression levels were quantified, and the read count matrix was normalized using variance stabilizing transformation (VST) in *DESeq2*.

### Read coverage plots of sequencing data

Per-base sequencing coverage across viral genomes was calculated using the *samtools depth* function. To enable cross-sample comparison of viral expression, coverage values were normalized to account for variations in sequencing depth. Normalization factors were determined using the *estimateSizeFactors* function from the *DESeq2* package, which adjusts for library size differences by estimating scaling factors for each sample. The normalized coverage profiles were then visualized using the *ggplot2* package.

### Public transcriptomic data acquisition and selection

To complement our *de novo* RNA-seq analysis, publicly available EBOV infection time-series data sets were obtained from the Gene Expression Omnibus (GEO) database. Data sets were subjected to a rigorous quality control and selection process to ensure biological relevance and data integrity. Our inclusion criteria were as follows: (i) Human cell model: EBOV infection experiments conducted in human cell lines, preferably Huh7, for consistency with our own studies. (ii) Temporal resolution: inclusion of multiple distinct time points to effectively capture dynamic transcriptional responses. (iii) Biological replicates: a minimum of three biological replicates per condition at each time point, crucial for statistical power. (iv) Data quality: initial assessment via PCA to confirm that time was the predominant driver of transcriptional variation, and to identify data sets with minimal batch effects or poor biological replicate clustering. Based on these criteria, we initially screened multiple data sets (GSE65573, GSE69942, GSE80058, GSE80832, GSE86539, and GSE210189). As detailed in Fig. S2, PCA revealed that GSE86539 and GSE210189 consistently met our stringent quality and experimental design standards, displaying clear time-driven separation of samples and robust clustering of biological replicates. Specifically, GSE86539 utilized Huh7 cells, which align with our own experimental model, and was thus selected for in-depth comparative transcriptomic analysis to identify infection-specific co-expression modules. GSE210189, profiling HUVEC cells, was used to cross-validate gene expression patterns. Conversely, other data sets were excluded: GSE65573 had insufficient biological replicates (e.g., only two at several time points); GSE69942 exhibited prominent batch effects; and GSE80058 and GSE80832 showed poor biological reproducibility with replicates failing to cluster cohesively. This rigorous selection process ensures that our integrated analyses are based on high-quality, biologically relevant public data, enhancing the robustness and interpretability of our findings.

### Microarray analysis

Raw intensity data from the Agilent-026652 Whole Human Genome Microarray 4x44K v2 were extracted using the read.imagene function from the *limma* package. Probe annotations were mapped to gene symbols based on the GEO platform GPL13497. Background correction was performed using the *backgroundCorrect* function with the “normexp” method to reduce systematic noise. Normalization across arrays was applied using the *normalizeBetweenArrays* function with the “quantile” method to enhance data comparability. Differential gene expression analysis was conducted using the *lmFit* function for linear model fitting, followed by *makeContrasts* and *contrasts.fit* to specify and apply contrast matrices for statistical inference.

### Bioinformatic analysis

Differential expression analysis was performed on each omics data set using the above RNA sequencing pipelines and data management protocols. PCA was applied to the entire feature set to delineate the primary sources of variation, with group contributions quantified via the *FactoMineR* and *DEGreport* packages. Unsupervised hierarchical clustering using the *pheatmap* package enabled the identification of global expression patterns across samples. The fuzzy c-means algorithm implemented in *Mfuzz* was employed to cluster features with similar temporal expression profiles. GO and Kyoto Encyclopedia of Genes and Genomes (KEGG) annotations for Homo sapiens were obtained from *g:Profiler* (https://biit.cs.ut.ee/gprofiler/gost), and subsequent enrichment and pathway analyses were performed using the *enricher* function in the *clusterProfiler* package with a significance cutoff of *P* < 0.05. Set intersections were systematically analyzed with the *VennDetail* package, and overlapping gene sets were visualized using *UpSetR*. Finally, temporal variations in gene expression between virus-infected and control samples were examined via the *ComplexHeatmap* package.

### Causal structure inference

To delineate the sequential activation of genes following viral infection, we employed causal structure inference (*CSI, https://github.com/cyversewarwick/csi*) on our time-series expression data. CSI infers directional regulatory interactions by evaluating whether the expression trajectory of one gene predicts subsequent changes in another, accounting for inherent delays in transcription, translation, and protein translocation. Mean expression profiles of differentially expressed genes, aggregated within each *Mfuzz* cluster, were used as input for the CSI analysis. The resultant regulatory network was visualized using *Cytoscape*.

### Drug repurposing and cheminformatics analysis

To identify host factors that interact with viral proteins, we extracted hub genes from the HVIDB, which comprises 48,643 experimentally validated human-virus protein-protein interactions (60). Candidate antiviral compounds were prioritized by integrating these hub genes with gene-drug associations from DGIdb, using the *queryDGIdb* function in the *rDGIdb* R package. A comprehensive virus-gene-drug interaction network was then constructed and visualized with the *ggraph* package. Chemical structures and physicochemical properties of the candidate compounds were retrieved using the *webchem*, *ChemmineR*, and *openbabel* packages. Molecular fingerprints were computed using the *get.fingerprint* function from the *rcdk* package, and pairwise Tanimoto similarity coefficients were calculated with the *fp.sim.matrix* function from the R *fingerprint* package. Hierarchical clustering was subsequently performed using *hclust* to identify structurally related antiviral candidates.

### siRNA treatment

All siRNAs used in this study were designed and synthesized by Sangon Biotech (Shanghai, China). The corresponding catalog numbers are listed in the revised Table S6. In six-well plates, 2 × 10⁵ cells per well were seeded and incubated overnight for adherence. The following day, siRNAs targeting specific host genes (40 nM final concentration) and a non-targeting scramble control were each diluted in Opti-MEM (Gibco, cat. no. 31985-070) according to the manufacturer’s instructions. These diluted siRNAs were then combined with RNATransMate (Sangon Biotech, cat. no. E607402) at a 1:1 (vol/vol) ratio and incubated at room temperature for 10 minutes to form transfection complexes. Cells were rinsed with serum-free DMEM before adding the transfection complexes. After 4 hours of incubation, the medium was replaced with antibiotic-free complete DMEM. Cells were maintained at 37 °C in a humidified atmosphere with 5% CO₂ for 48–72 hours. To assess knockdown efficiency, total RNA was extracted as previously described, and qRT-PCR was performed using gene-specific primers. To evaluate the impact of siRNA-mediated gene silencing on viral replication, cells were infected with the virus at an MOI of 1, 24 hours post-transfection. Viral RNA levels in both cell lysates and supernatants were quantified by RT-qPCR at 24 and 48 hours post-infection. All experiments were conducted in triplicate to ensure reproducibility.

### Western blot analysis

Huh7 cells were transfected with target-specific or non-targeting control siRNAs. At 72 hours post-transfection, cells were lysed in RIPA buffer containing protease inhibitors. Equal protein amounts (20 μg) were separated by SDS-PAGE, transferred to PVDF membranes, and probed with primary antibodies against LDLR, AHR, RELB, c-MYC, QKI, and β-Tubulin (loading control), followed by HRP-conjugated secondary antibodies and ECL detection.

### Analysis of the drug cytotoxicity

Huh7 cells were seeded in 96-well plates at a density of 1 × 10⁴ cells per well and incubated overnight at 37 °C in 5% CO₂ to allow adherence. The following day, cells were treated in triplicate with serial dilutions of the test compounds, including 6α-Methylprednisolone 21-hemisuccinate sodium salt (MedChemExpress, CAS 2375-03-3), calcitriol (Macklin, CAS 32222-06-3), favipiravir (YUANYE, CAS 259793-96-9), verapamil (Solarbio, CAS 52-53-9), resveratrol (GLPBIO, CAS 501-36-0), sorafenib (APExBIO, CAS 284461-73-0), and thioguanine (Sigma-Aldrich, CAS 154-42-7), prepared in DMEM supplemented with 10% FBS. After a 48-hour incubation under the same conditions, the medium was replaced with fresh DMEM containing 10% FBS and 10 μL of Cell Counting Kit-8 (CCK-8) solution (Vazyme, cat. no. A311-01), following the manufacturer’s instructions. Cells were then incubated for 3 hours at 37 °C in 5% CO₂. Absorbance at 450 nm was measured using a Fluostar Omega Plate Reader (BMG Labtech) to assess cell viability. The highest non-toxic concentration of each drug was determined for subsequent experiments. In cases where all tested concentrations exhibited cytotoxicity, the lowest concentration was selected. All experiments were performed in triplicate to ensure reproducibility.

### Drug treatment

Huh7 cells were treated with each compound at a defined concentration or 1% (vol/vol) dimethyl sulfoxide (DMSO) as a control for 2 hours prior to EBOV infection or treated 2 hours post-infection. Cells were infected with EBOV at an MOI of 0.1 and incubated at 37 °C in 5% CO₂. At 24 hours, 48 hours, and 72 hours post-infection, RNA was extracted from both cell lysates and supernatants as described above. Viral RNA levels were quantified by RT-qPCR. The dose-dependent antiviral effect of each compound was evaluated based on viral RNA quantification. The half-maximal effective concentration (EC50) was calculated using a four-parameter log-logistic regression model (“LL.2”) in the R package *drc*.

### Statistical analysis

All statistical analyses were conducted in R. Group comparisons were performed using Student’s t-test. A *P*-value of less than 0.05 was deemed statistically significant. Significance levels were annotated as follows: (*) *P* ≤ 0.05; (**) *P* ≤ 0.01; (***) *P* ≤ 0.001; (****) *P* ≤ 0.0001; non-significant differences were labeled as “ns.” Additionally, Fisher’s exact test was applied to evaluate the significance of overlap between modules.

## Data Availability

The RNA-seq data of this study have been deposited in the Genome Sequence Archive (GSA) (https://bigd.big.ac.cn/) with BioProject number: PRJCA038368 and Accession number: HRA011041. All custom analysis scripts and workflows used to generate the results presented in this manuscript are freely available in the Zenodo repository (DOI: 10.5281/zenodo.19123964).

## SUPPLEMENTAL MATERIAL

Fig. S1 spectrum.03608-25-s0001.tif Biological process terms related to immunity and viral response in co-expression modules derived from RNA-seq data.

Fig. S2 spectrum.03608-25-s0002.tif PCA-based quality assessment and selection of public EBOV transcriptomic data sets.

Fig. S3 spectrum.03608-25-s0003.tiff Protein-level validation of siRNA knockdown by Western blotting.

Supplemental material spectrum.03608-25-s0004.docx Supplemental table and figure legends.

Table S1 spectrum.03608-25-s0005.xlsx Gene expression matrix and differentially expressed genes across time points in EBOV-infected Huh7 cells (RNA-seq).

Table S2 spectrum.03608-25-s0006.xlsx Co-expression modules identified by Mfuzz in EBOV-infected Huh7 cells.

Table S3 spectrum.03608-25-s0007.xlsx Gene expression matrix and differentially expressed genes from EBOV-ΔVP30-infected Huh7 cells (GSE86539 data set).

Table S4 spectrum.03608-25-s0008.xlsx Co-expression modules identified by Mfuzz in EBOV-ΔVP30-infected Huh7 cells (GSE86539 data set).

Table S5 spectrum.03608-25-s0009.xlsx Initial drug screening concentrations and minimum cytotoxic concentrations.

Table S6 spectrum.03608-25-s0010.xlsx Details of double-stranded siRNAs employed in this study.

ASM does not own the copyrights to Supplemental Material that may be linked to, or accessed through, an article. The authors have granted ASM a non-exclusive, world-wide license to publish the Supplemental Material files. Please contact the corresponding author directly for reuse.
