# Supplementary figures and images for: Temporal transcriptomics identifies early-response and infection-condition-specific modules guiding host-directed anti-EBOV therapeutics
Source: Microbiol Spectr. 2026 Apr 30;14(6):e03608-25. doi: 10.1128/spectrum.03608-25 (PMC13228025; doi:10.1128/spectrum.03608-25)

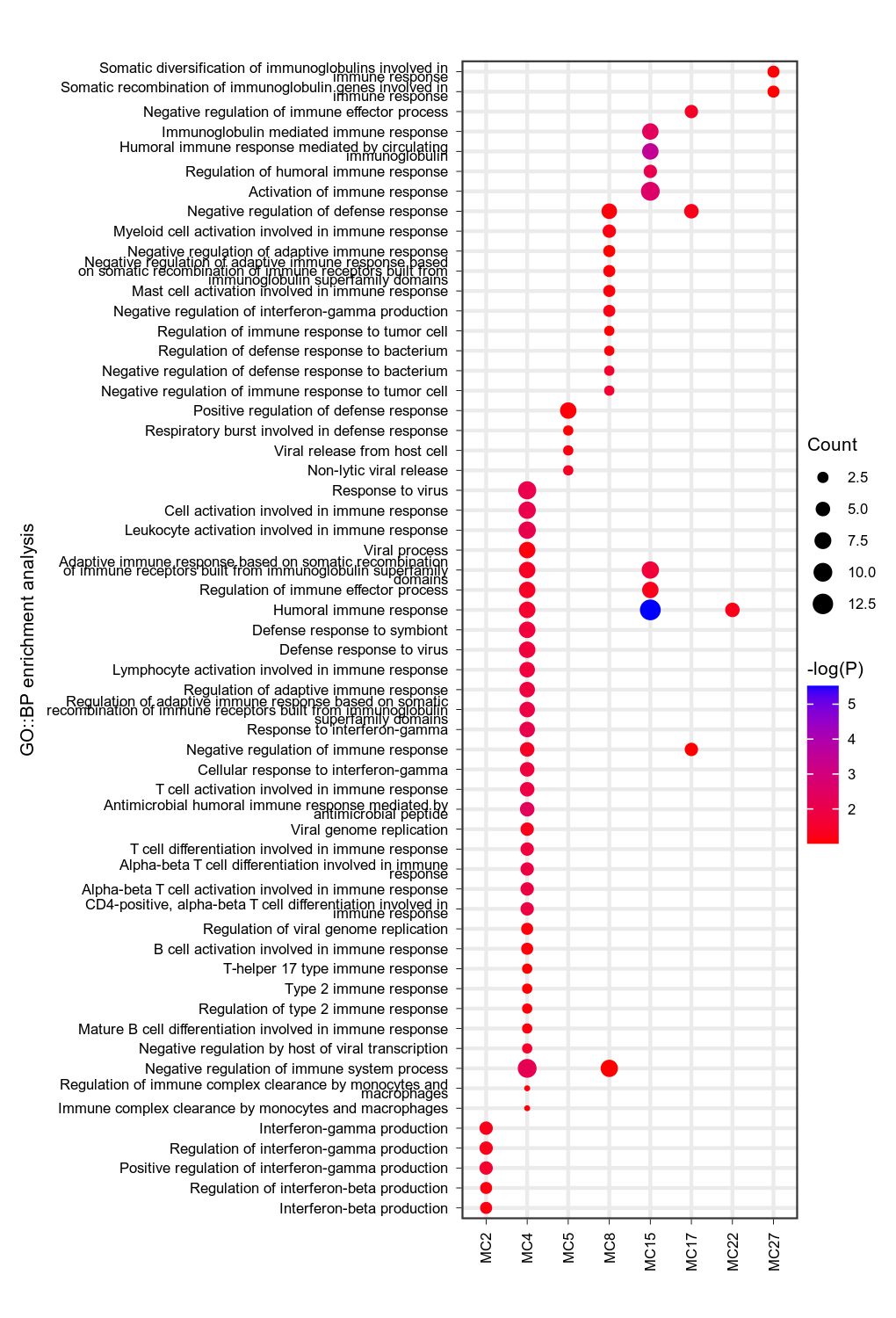

Supplement: Fig. S1 — Biological process terms related to immunity and viral response in co-expression modules derived from RNA-seq data. [file spectrum.03608-25-s0001.tif]

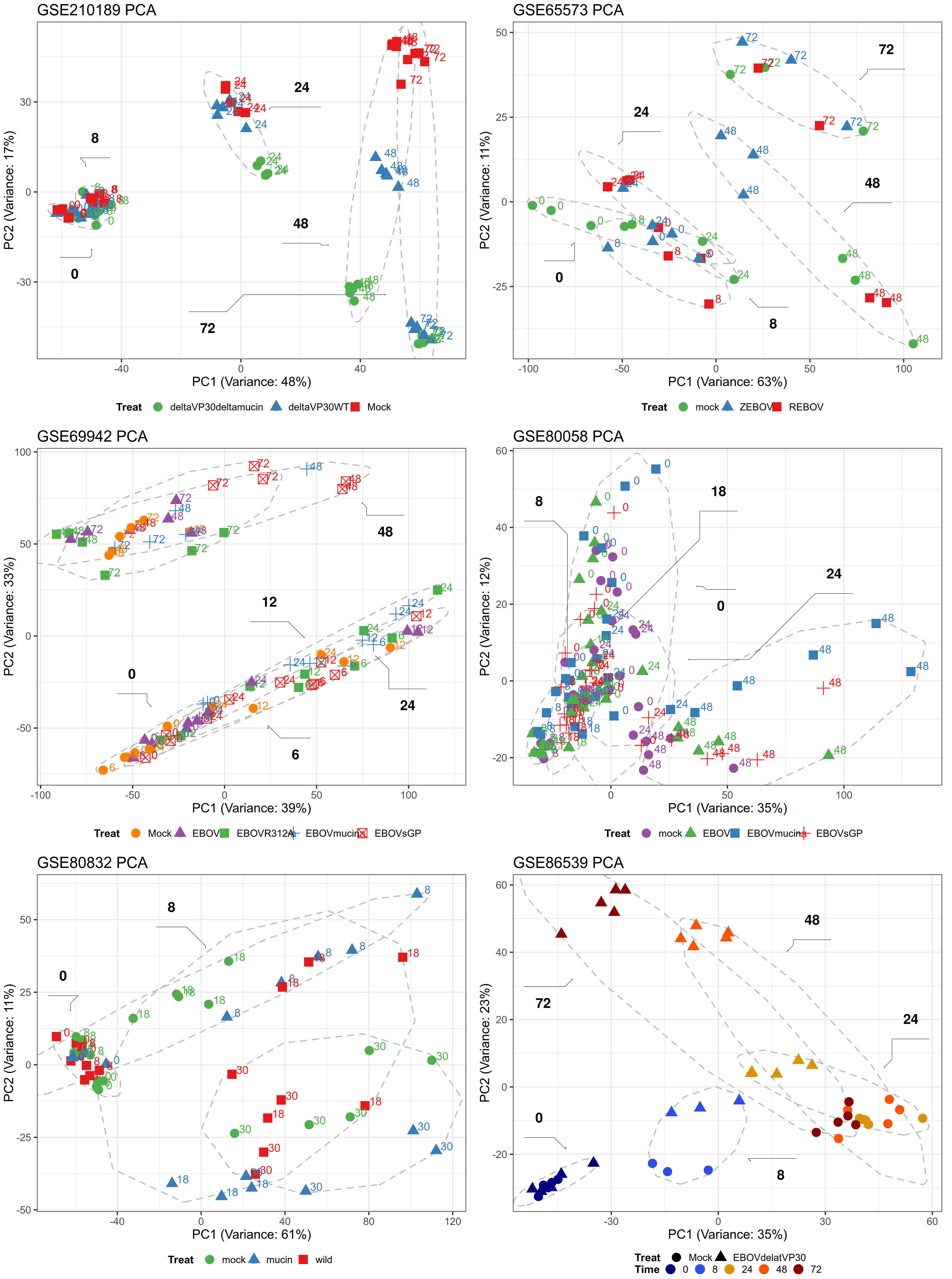

Supplement: Fig. S2 — PCA-based quality assessment and selection of public EBOV transcriptomic data sets. [file spectrum.03608-25-s0002.tif]

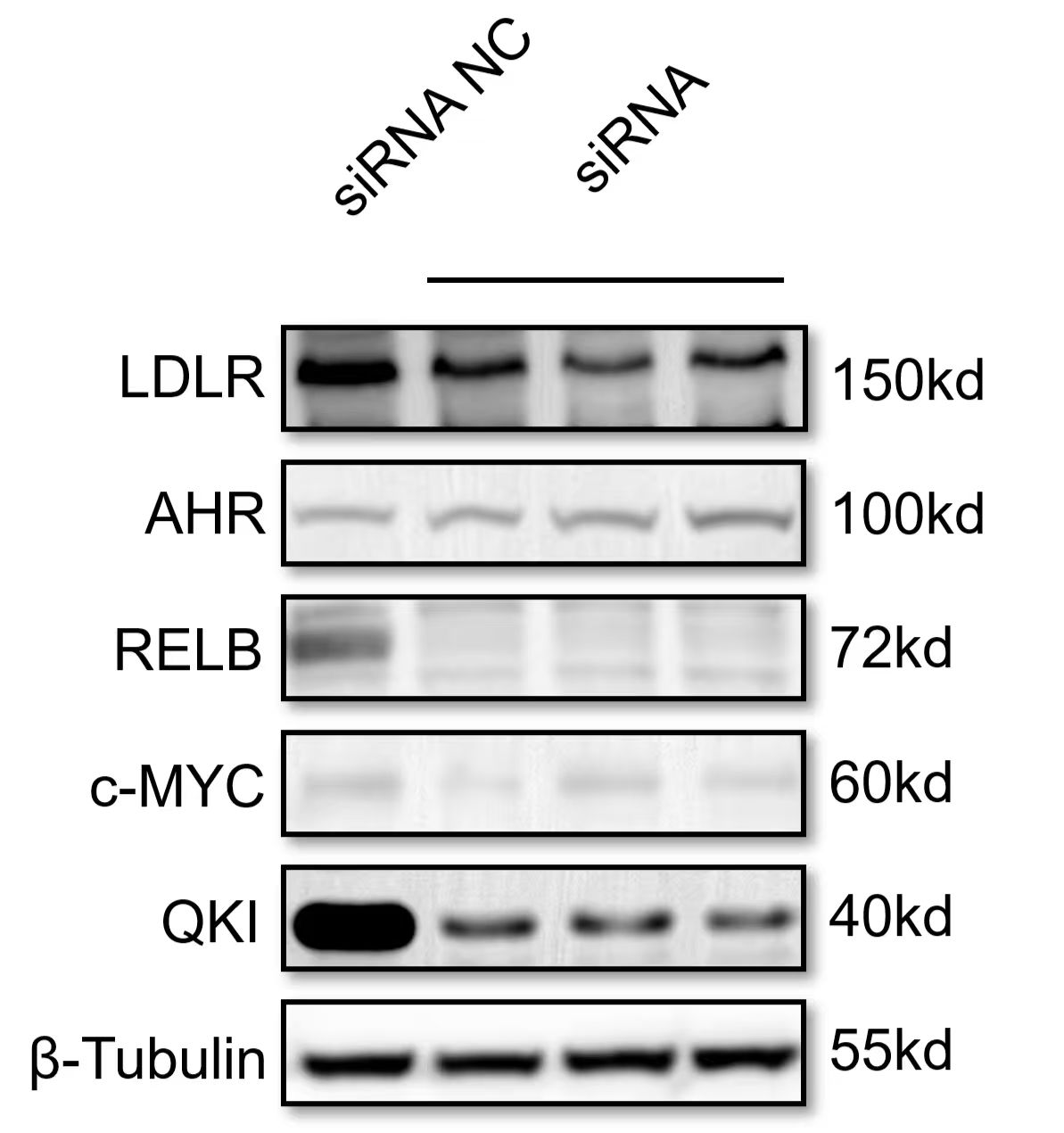

Supplement: Fig. S3 — Protein-level validation of siRNA knockdown by Western blotting. [file spectrum.03608-25-s0003.tiff]
