# Supplementary material for: Temporal transcriptomics identifies early-response and infection-condition-specific modules guiding host-directed anti-EBOV therapeutics
Source: Microbiol Spectr. 2026 Apr 30;14(6):e03608-25. doi: 10.1128/spectrum.03608-25 (PMC13228025; doi:10.1128/spectrum.03608-25)
Supplement: Supplemental material — Supplemental table and figure legends. [file spectrum.03608-25-s0004.docx]

**Supplementary information**

**Supplementary figure**

**Supplementary Figure S1 | Enriched GO: Biological Process terms related to immunity and viral response in co-expression modules derived from RNA-seq data.**

**Supplementary Figure S2: PCA-based quality assessment and selection of public EBOV transcriptomic datasets.** PCA plots show transcriptional variance in six public EBOV infection datasets (GSE210189, GSE65573, GSE69942, GSE80058, GSE80832, and GSE86539) from GEO. Each point represents a biological replicate, colored by treatment and shaped by time post-infection (hpi). Dashed lines delineate time points.

**Supplementary Figure S3.** Protein-level validation of siRNA knockdown by Western blotting. Huh7 cells were transfected with siRNAs targeting RELB, LDLR, AHR, c-MYC, QKI, or non-targeting control (NC). At 72 h post-transfection, whole-cell lysates were analyzed. β-Tubulin served as loading control. Knockdown was robust for RELB and QKI and partial for LDLR, AHR, and c-MYC. Molecular weights are indicated on the right (kDa).

**Supplementary tables**

**Supplementary Table S1** | Gene expression matrix and differentially expressed genes across time points in EBOV-infected Huh7 cells (RNA-seq).

**Supplementary Table S2** | Co-expression modules identified by Mfuzz in EBOV-infected Huh7 cells.

**Supplementary Table S3** | Gene expression matrix and differentially expressed genes from EBOV-ΔVP30-infected Huh7 cells (GSE86539 dataset).

**Supplementary Table S4** | Co-expression modules identified by Mfuzz in EBOV-ΔVP30-infected Huh7 cells (GSE86539 dataset).

**Supplementary Table S5** | Initial drug screening concentrations and minimum cytotoxic concentrations.

**Supplementary Table S6** | Details of double-stranded siRNAs employed in this study.
